# Regional disparities in health care resources in traditional Chinese medicine county hospitals in China

**DOI:** 10.1371/journal.pone.0227956

**Published:** 2020-01-21

**Authors:** Dawei Zhu, Xuefeng Shi, Stephen Nicholas, Ping He

**Affiliations:** 1 China Center for Health Development Studies, Peking University, Beijing, China; 2 School of Management, Beijing University of Chinese Medicine, Beijing, China; 3 National Institute of Chinese Medicine Development and Strategy, Beijing University of Chinese Medicine, Beijing, China; 4 Research Institute for International Strategies, Guangdong University of Foreign Studies, Guangdong, China; 5 School of Economics and School of Management, Tianjin Normal University, Tianjin, China; 6 TOP Education Institute, Sydney, New South Wales, Australia; 7 Newcastle Business School, University of Newcastle, Newcastle, New South Wales, Australia; China Agricultural University, CHINA

## Abstract

**Objective:**

We aimed to analyze regional disparities of health care resources in traditional Chinese medicine (TCM) county hospitals and their time trends, and to assess the changes of regional disparities before and after 2009 health care reforms.

**Methods:**

We used hospital-based, longitudinal data from all TCM county hospitals in China between 2004 and 2016. To measure the key development features of TCM county hospitals, data were collected on government hospital investment, hospital numbers (the average number of TCM hospitals per county), hospital scale (the number of medical staff and hospital beds) and doctors’ workload (the daily visits and inpatient stays per doctor). We used segmented linear regression to test the time trend for outcome variables. We set a breakpoint at 2011, dividing the pre-reform (2004–2011) and post-reform (2012–2016) periods.

**Results:**

After the 2009 health reforms, TCM hospitals continued to display large disparities in the number, scale, and doctors’ workload across the three regions. In the pre-reform period, yearly government subsidies for TCM hospitals in western area were roughly RMB0.6 million (US$89 thousand) more than those in central and eastern region, which increased under the 2009 reforms to roughly RMB2 million (US$298 thousand) more per yer in post-reform period. These increased subsidies saw an increase in the number of TCM hospitals in the western area, partly addressing regional disparities. But there was no improvement in the regional disparities in terms of scale (number of beds) and the doctors’ workload (daily outpatient visits and inpatients per doctor) increased or remained unchanged between the western and other regions.

**Conclusion:**

Although TCM hospital number, scale, and doctors’ workload increased over the past 13 years, substantial regional disparities remained. The 2009 health reforms did not significantly change the regional disparities in health care resources, especially between the eastern and western regions.

## Introduction

Regional disparities in health care is a global phenomenon, and a persistent feature of China’s health care system [1]. Regional disparities describe the differences in the character and process of health care and variations in health care facilities across different regions, which leads to health welfare inequalities within a country [2]. The equitable allocation of health care resources ensures fairness in health care accessibility, but inequalities in health care resources create or exacerbate disparities in both health system quality and health outcomes [3–5]. Similar to geographical disparities in China’s economic development, provinces in western and central China have much lower densities of health facilities, health workers and hospital beds compared with more developed eastern provinces [6–8]. These disparities increased between 1980 and 2003, where the absolute gap in beds per thousand people among eastern, central and western and regions increased [8].

In China, the health system can be divided into a western and traditional Chinese medicine (TCM) system. TCM hospitals (including ethnic minority medicine hospitals and integrated Chinese and Western medicine hospitals TCM hospitals) were the main Chinese medicine providers that treat the patients with TCM service and products (including medicinal herbs, moxibustion, acupuncture, dietary therapy, massage and therapeutic mind/body practices) to maintain public health[9,10]. Given China’s cultural heritage, the promotion of TCM has always been a priority in China, with the demand for TCM services increasing in recent years [11]. Since 1954, TCM hospitals were initiated by the central government, and treat patients with TCM service and medicine[12]. During 1970s, the China government proposed the policy of “integration of Chinese and western medicine”, and more and more modern medicine and technologies were introduced to TCM hospitals[12].

Currently, county level TCM hospitals were the main providers of TCM services and also principal providers of western medical services, especially in rural areas [12]. The number and scale of TCM county hospitals played a significant role in assuring equitable distribution of health care resources and guaranteeing health care accessibility. In spite of government support for TCM, TCM hospitals were no exception to the regional inequality in the distribution of China’s health care resources, with the eastern region having higher beds counts and doctor density than western or central areas [11].

Prompted by the 2003 SARS epidemic, Chinese governments recognized the fragility and inequalities of health system, taking the first tentative strategies to balance health care resources across regions. The majority of government investments in health care come from local government, therefore the unequal financial capacity of provinces to invest in health care, a reflection of China’s unbalanced economic development, explains much of the regional health resource disparities [8]. To address these regional disparities, the central government increased transfer payments to the central and western provinces [13] to improve equality of the health system, with the establishment of many new health facilities [14].

In 2009, China launched a new round of health system reform [15], aiming to provide equitable, affordable and effective basic health care for all households by 2020 [16]. To achieve these goals, funding was directed to building health infrastructure, training primary health care providers, subsidizing enrollment in basic health insurance schemes, and providing public health facilities [16]. With regional health service equality a continuing basic principle of the health reform agenda, the 2009 reforms addressed the imbalances in health care availability and access across regions, and implemented at county hospitals since 2012 [17,18]. In terms of equality in health care resource, the central government strengthened their investment in county hospitals and grassroots township and village health care institutions [19], including further reinforcing the system of TCM [20,21]. Did the 2009 health funding reforms close the gap in the regional disparities in TCM health care availability and access?

In addition, China’s State Council issued an outline of strategic planning for the development of TCM in 2016, which pointed out that the supply of TCM services did not meet the people’s need due to limited TCM healthcare resources and the availability of services and therefore set the goal of universal access to TCM services by the end of 2020[9]. Exploring the distribution of TCM health resources and effectively improving the equitable distribution of these resourcesis are critical to achieving universal access to TCM services. Although scholars have been interested in the regional disparities in health care resource distribution [6,7,22–24], there are few studies of the changing regional disparities in TCM health resources [11]. Since hospitals occupied major health resources and were the main health care providers in China [7], understanding the changes in the regional disparity in hospital resources provides general insights in health care resource allocation. In the context of the 2009 health care reform and the outline of strategic planning for the development of TCM, this paper explores whether the regional disparities in TCM county hospitals improved, remained the same or deteriorated.

Using longitudinal data from all TCM county hospitals in China between 2004 and 2016, we aimed to analyze the changes in regional disparities, and their time trends, in terms of their number, scale, and doctor’s workload. Further, we assessed whether the tendency of those regional disparities differed before and after the 2009 health care reforms. Since differentiated government investment is the main policy instrument for promoting the development of TCM county hospitals, we also evaluated the regional disparities in government hospital investment.

## Methods

### Data source

Our study used a unique hospital-based, longitudinal dataset from all TCM county hospitals (including ethnic minority medicine hospitals and integrated Chinese and Western medicine hospitals) in China between 2004 and 2016. Collected annually by China’s National Health Commission, these longitudinal hospital data cover the period before and after the implementation of China’s 2009 health system reforms, comprising information on health resources, revenues, and outpatient and inpatient services.

### Measurements

We used the total government subsidy per hospital per year to measure government investment in TCM county hospitals. To measure the key features of TCM county hospitals, we used data on number (the average number of TCM hospitals per county), scale (the number of medical staff and hospital beds) and doctor’s workload (daily visits per doctor and inpatient stays per doctor). During the Seventh Five-Year Plan period (1986–1990), the Chinese government divided its 31 provinces on the mainland into three regions according to their geographical location and level of economic development, and this division was wildy used in previous researches.[11,25–27]Follow this division, we divided the 31 provinces, autonomous regions and municipalities into three regions according to their geographical location. The eastern region comprised 11 provinces (Beijing, Fujian, Guangdong, Hainan, Hebei, Jiangsu, Liaoning, Shandong, Shanghai, Tianjin, and Zhejiang); the central region included 8 provinces (Anhui, Heilongjiang, Henan, Hubei, Hunan, Jiangxi, Jilin, and Shanxi); and the western region included 12 provinces (Chongqing, Gansu, Guangxi, Guizhou, Inner Mongolia, Ningxia, Qinghai, Shaanxi, Sichuan, Tibet, Xinjiang, and Yunnan).

### Statistical analysis

Initially, we performed descriptive analysis. We then tested whether there was a statistically significant change in the time trend in the outcome variables compared with the period before and after the health system reform using segmented regression models. The segmented regression model was specified as follows:

$$Y_{it}=\beta_{0}+\beta_{1}Region+\beta_{2}Eastern*t_{1}+\beta_{3}Central*t_{1}+\beta_{4}{Western*t}_{1}+\beta_{5}{Eastern*t}_{2}+\beta_{6}Central*t_{2}+\beta_{7}Western*t_{2}+\varepsilon_{it}$$ (1)

where *Y*_*it*_ represents the outcome variables in hospital (*i*) and year (*t*); *Region* is a categorical variable indicating whether an hospital is within eastern, central, or western regions; *Eastern*, *Central*, and *Western* are three indicator variable to denote the eastern, central, and western regions; it *t*_1_ is annual trend term for the before reform period; *t*_2_ is annual trend term for the after reform period; *ε*_*it*_ refers to the error term. This segmented regression model provided estimations of the time trends in the before period (*β*_2_ to *β*_4_) and whether there was a statistically significant change in this time trend between periods (*β*_5_ to *β*_7_). According to model 1, we can also calculate the trend in the absolute gap between eastern central and western region using *β*_2_ − *β*_4_ (eastern vs western) and *β*_3_ − *β*_4_ (central vs western), respectively, and whether there was a statistically significant change in this trend between periods using *β*_5_ − *β*_7_ (eastern vs western) and *β*_6_ − *β*_7_ (central vs western) [28].

The reform policy was implemented progressively, and it was implemented on TCM hospitals sicne 2012, which makes it impossible to determine *a priori* at which time points we might expect the time trends to change. We used an iterative search procedure to identify which breakpoint provided the best fit for the data by comparing all models using different 2009–2013 alternative breakpoints[29]. Our results indicated that the breakpoint at 2011 provided the best fit for the data (S1 Fig). The two segments were divided at 2011, pre-reform (2004–2011) and post-reform (2012–2016), and we then used the breakpoint at 2011 in all further models. The software Stata version 15 for Windows (Stata Corp, College Station, TX, USA) was used for the statistical analysis.

### Ethics statement

The study collated secondary data collected annually by the National Health Commission of the People’s Republic of China, and contained no individual-specific information. Hence ethical approval was not required.

## Results

### Characteristics of TCM hospitals

The impact of increased government health facility investment was reflected in the increase in county level TCM hospitals from 1488 in 2004 to 1743 in 2016. As shown in Table 1, in the pre-reform period, the average annual government subsidy per hospital in the western region was RMB1.8 million (US$268 thousand), roughly RMB0.5 million (US$74 thousand) more than the central and RMB0.7 million (US$104 thousand) more than the eastern region. In the post-reform period, government subsidies rose in all regions, but especially in the western region. The gap in funding between western and other regions roughly doubled to RMB2 million (US$298 thousand), indicating that government investment was directed to improving regional disparities in TCM hospital availability. Table 1 also reports that there remained more county level TCM hospitals in each county in the eastern region than the central and western regions. Hospitals in the eastern region also had greater scale, measured by more medical staff and more hospital beds, than hospitals in the western region, and a greater scale than central TCM hospitals measured by the number of medical staff. As shown in Table 1, hospitals in the western region had the highest doctor’sworkload, with more daily visits by doctors and more inpatient stays per doctor; hospitals in the central region displayed medium scale and doctor’s workload compared to eastern region hospitals.

**Table 1:** Characteristics of TCM hospitals, 2004–2016.

	2004–2011			2012–2016		
	Eastern Region	Central Region	Western Region	Eastern Region	Central Region	Western Region
Government subsidy (Million Yuan)	1.10(2.48)	1.27(2.33)	1.80(3.37)	3.90(6.92)	4.15(5.84)	6.06(7.80)
Average number of TCM hospitals per county	1.17(0.04)	1.13(0.02)	1.08(0.01)	1.28(0.03)	1.24(0.06)	1.21(0.06)
Number of medical staff	133.68(84.40)	127.35(88.87)	78.92(59.72)	184.29(144.66)	176.74(151.91)	121.88(107.30)
Number of hospital beds	113.46(77.32)	107.26(82.95)	84.44(65.65)	184.22(147.64)	191.29(174.49)	153.31(121.88)
Daily visits per doctor	6.03(4.35)	4.85(3.78)	7.29(8.73)	7.32(5.23)	5.63(4.63)	7.44(5.18)
Daily inpatient stays per doctor	1.33(0.97)	1.42(1.61)	1.73(1.94)	2.06(1.21)	2.43(1.80)	3.03(2.02)
Observations (Hospital-year)	2622	3762	4773	1859	2703	3426

### Average changes in the characteristics of TCM hospitals

**Fig 1:**
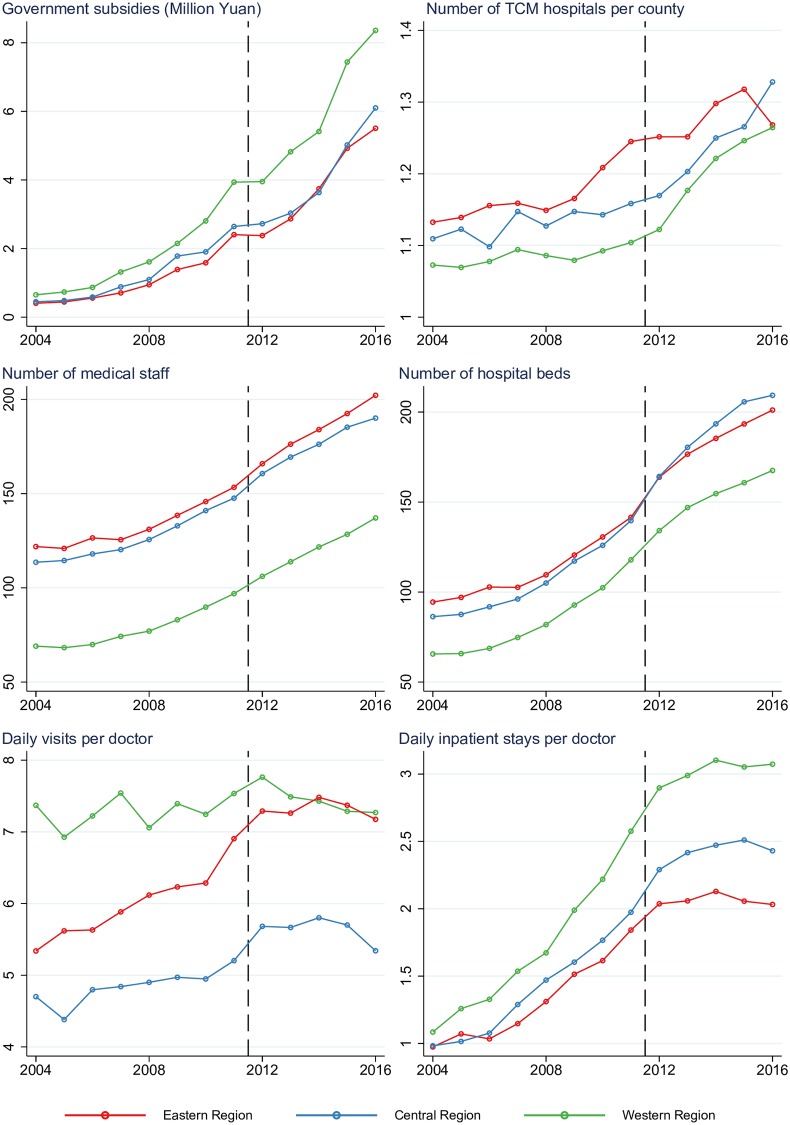
Year-by-year changes in the characteristics of TCM hospitals, 2004–2016.

Fig 1 presents the year-by-year trends in the key characteristics of TCM hospitals, and Table 2 illustrates the average changes in the key characteristics by period. In the pre-reform period, the government subsidy per hospital increased RMB443 thousand (US$66 thousand) per year in the western region and about half that rate, RMB275 thousand (US$41 thousand) per year, in the eastern and central regions. This trend continued in the post-reform period, where the government subsidy increased by RMB691 thousand (approximately US$103 thousand) in western region, outpacing the RMB550 thousand (approximately US$82 thousand) increase in eastern and central regions. As shown in Fig 1, increasing trends were also observed in average number and scale of county level TCM hospitals in all three regions in both periods. The rate of increase in the average number of hospitals was significantly higher in the post-reform period than the pre-reform period in the central and western regions, with no significant change in the eastern region (see Table 2). There was a significant increase in the rate of increase in the number of medical staff and a significant decrease in the rate of increase in inpatient stays in all regions. The eastern and central areas witnessed accelerated growth in hospital beds after 2011. In all three regions, daily outpatient visits per doctor increased only before 2012.

**Table 2:** Estimated average changes in the characteristics of TCM hospitals, 2004–2016.

		Annual change in outcomes			Change in trend of outcomes from previous period		
		Eastern	Central	Western	Eastern	Central	Western
Government subsidy (Million Yuan)	2004–2011	0.266(0.032)***	0.287(0.027)***	0.443(0.024)***			
	2012–2016	0.823(0.066)***	0.831(0.054)***	1.134(0.049)***	0.556(0.089)***	0.544(0.073)***	0.691(0.065)***
Average number of TCM hospitals per county	2004–2011	0.016(0.002)***	0.007(0.002)**	0.007(0.002)**			
	2012–2016	0.014(0.004)**	0.039(0.004)***	0.041(0.004)***	-0.002(0.006)	0.032(0.006)***	0.034(0.006)***
Number of medical staff	2004–2011	5.826(0.679)***	6.164(0.567)***	4.977(0.503)***			
	2012–2016	10.89(1.398)***	9.364(1.151)***	9.086(1.028)***	5.065(1.871)**	3.200(1.549)*	4.109(1.383)**
Number of hospital beds	2004–2011	8.884(0.724)***	10.256(0.604)***	9.331(0.536)***			
	2012–2016	12.860(1.490)***	15.66(1.226)***	11.06(1.096)***	3.979(1.994)*	5.399(1.65)**	1.730(1.474)
Daily visits per doctor	2004–2011	0.238(0.039)***	0.134(0.032)***	0.056(0.029)*			
	2012–2016	0.062(0.08)	0.022(0.066)	-0.078(0.059)	-0.176(0.107)	-0.113(0.089)	-0.135(0.079)
Daily inpatient stays per doctor	2004–2011	0.143(0.011)***	0.175(0.009)***	0.236(0.008)***			
	2012–2016	0.029(0.023)	0.081(0.019)***	0.088(0.017)***	-0.114(0.03)***	-0.094(0.025)***	-0.147(0.022)***

Standard errors in parentheses.

*** p<0.001,

** p<0.01,

* p<0.05.

### Trend in absolute gap in the characteristics of TCM hospitals

**Fig 2:**
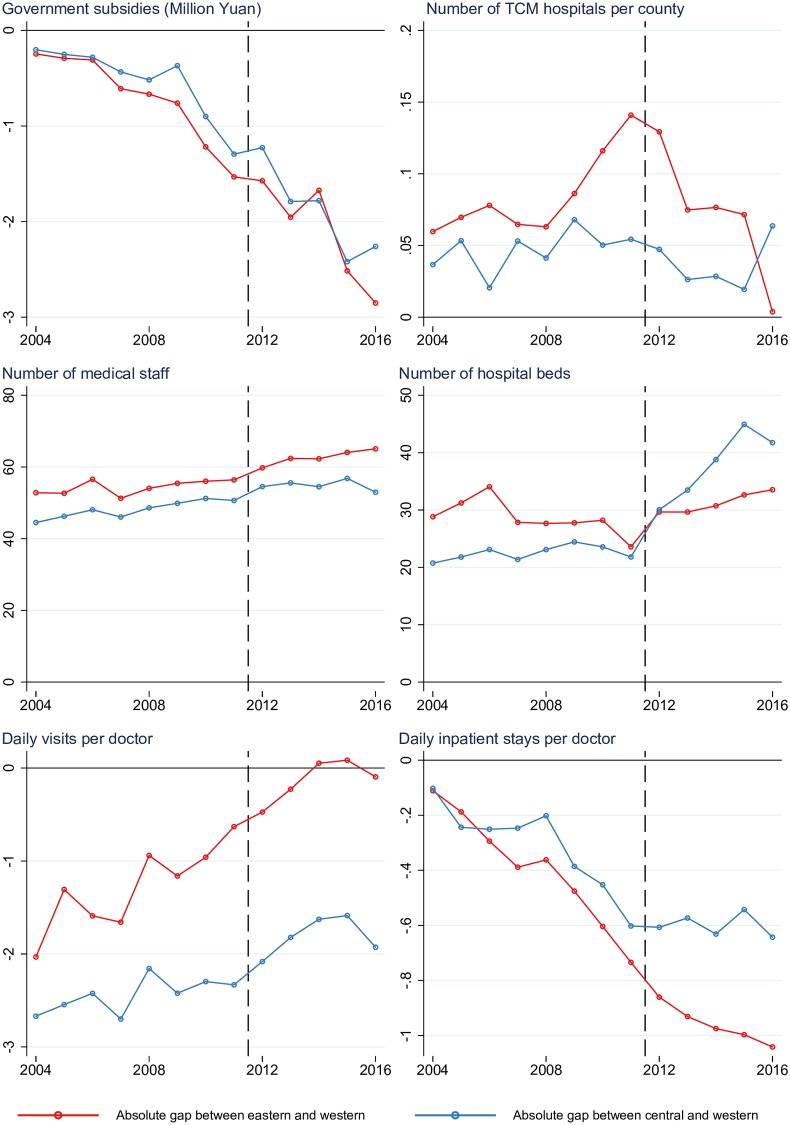
Year-by-year changes in the absolute gap in the characteristics of TCM hospitals, 2004–2016.

Fig 2 presents the year-by-year trends in the absolute gap in the key characteristics of TCM hospitals, and Table 3 estimates the average changes in the absolute gap in the two periods. The annual gap in government subsidy between eastern and western area was RMB176 thousand (US$26 thousand) and that between central and western area was 156 thousand (US) during the pre-reform period. There two gaps both rose to RMB300 thousand (US$45 thousand) during the post-reform period. The absolute gap in the average number of TCM hospitals per county between eastern and western region increased at a rate of 0.009 (*P =* 0.003*)* per year during the pre-reform period, but the regional west-east disparity in the number of hospitals per county narrowed in the post-reform period, decreasing at a rate of -0.027 (*P*<0.001) per year after 2011. The absolute gap in average number of TCM hospitals per county between the central and western region fluctuated around 0.05 in both periods, with no significant widening or narrowing of the disparity.

**Table 3:** Trend in absolute gap in the characteristics of TCM hospitals, 2004–2016.

		Annual change in absolute gap		Change in trend of absolute gap from previous period	
		Eastern-Western	Central-Western	Eastern-Western	Central-Western
Government subsidy (Million Yuan)	2004–2011	-0.176(0.04) ***	-0.156(0.036) ***		
	2012–2016	-0.311(0.082) ***	-0.303(0.073) ***	-0.135(0.11)	-0.147(0.098)
Average number of TCM hospitals per county	2004–2011	0.009(0.003)**	0.000(0.003)		
	2012–2016	-0.027(0.006)***	-0.002(0.006)	-0.036(0.008)***	-0.002(0.008)
Number of medical staff	2004–2011	0.849(0.845)	1.187(0.758)		
	2012–2016	1.805(1.736)	0.278(1.543)	0.956(2.327)	-0.909(2.076)
Number of hospital beds	2004–2011	-0.447(0.901)	0.925(0.808)		
	2012–2016	1.802(1.85)	4.594(1.645) **	2.249(2.48)	3.669(2.213)
Daily visits per doctor	2004–2011	0.182(0.048) ***	0.078(0.043)		
	2012–2016	0.141(0.099)	0.100(0.088)	-0.041(0.133)	0.022(0.119)
Daily inpatient stays per doctor	2004–2011	-0.093(0.014) ***	-0.061(0.012) ***		
	2012–2016	-0.059(0.028) *	-0.007(0.025)	0.034(0.038)	0.053(0.034)

Standard errors in parentheses.

*** p<0.001,

** p<0.01,

* p<0.05.

For both periods, there was no significant annual changes in the absolute gap in medical staff and beds between the three regions, except the absolute gap in hospitals beds between the central and western regions increased 4.594 (*P* = 0.005) per year per hospital during the post-reform period. During the pre-reform period, the absolute gap in daily visits per doctor between the eastern and western region decreased 0.182 (*P*<0.001) per year, which narrowed the western-eastern doctors’ burden. After 2011, decreasing rates in the absolute gap of daily doctor visits among the three regions were not significant. However, the absolute gap in the daily inpatients per doctor widened between the western and other regions sharply every year during the pre-reform period and moderately increased during the post-reform.

## Discussion

Using hospital-based longitudinal data, our study analyzed the regional disparities, and their time trends, in the key charactristics of county level TCM hospitals in the context of China’s 2009 health system reforms. Although government subsidies increased, TCM hospitals continued to display large disparities in the number, scale, and doctors’ workload across the three regions.

There were fewer TCM hospitals with smaller scale in the western region than in the eastern and central regions. In the pre-reform period, hospitals in western China received higher government subsidies compared to hospitals in eastern China, offsetting the greater and more stable financial capacity of local governments in the wealthy eastern region to invest in health [13]. But the gap in the number of western versus eastern hospitals in the pre-reform period widened, which was only reversed during the reform period. There was no change in the gap between the number of western versus central TCM hospitals. There was also no change in the scale of western TCM hospitals compared to the other regions. Given the lower size and density of population in the western region [7], it is reasonable that hospitals in the western region had a relative smaller scale than eastern or central TCM hospitals. However, in consideration of its vast territory, there should be a greater number of hospitals in the western region to preserve equity and accessibility to health services. This only happended in the post-reform period for the western-eastern regions. Disparities in access to TCM hospitals in the western region were no significantly narrowed. Measured by inpatients stays per doctor and doctor visits, TCM county hospitals in the western region had a higher doctors’ workload than those in the central and eastern region. The doctors’ workload of TCM hospitals in the western area was similar to the regional disparity in general hospitals, where inpatient stays per doctor and doctor visits were also greater in the western region [30]. The 2009 reforms did not narrow regional disparities by addressing the doctors’ workload.

While increased central government funding benefited the western region hospitals pre and post-reform, much of the central government special transfer payments were used for infrastructure investment [18], with about RMB63 billion (US$9 billion) to support county hospitals and other grassroots level health facilities. Most of the investment was used to expand the scale, rather than to increase the number of TCM hospitals. Also, local governments increased their financial input into health, and the subsidy policy channeled funding into basic construction and equipment procurement, development of key subjects, funds for retirees in conformity with the state regulations concerned, subsidies for policy-related losses, and special grants for government-sponsored tasks, such as public health services. These government subsidies stimulated the expansion of hospital scale, in terms of beds and medical staff. In addition, before 2011, hospitals were subject to headcount quota system regulation which controlled total staffing [31,32]. Since 2011, the State Council and Ministry of Health of China issued a series of policy directives to weaken the headcount quota system and strengthen the recruitment autonomy of hospitals [32]. While these policies accelerated key features of TCM hospitals, increasing the scale over expansion in the number of hospitals, instead of addressing the regional disparity issue for the western region that needed more, not larger, hospitals. A further impact may have been an increase in doctors’ workload [33].

Favouring the western region, the absolute gap in government subsidy increased year by year due to the increased transfer payments since 2000, and especially after the 2009 reforms. The increased transfer payments can explain the reduced disparities in the number of TCM hospitals between the eastern and western regions after reform. There was no significant change in the disparities in medical staff between three regions in the two periods, but the disparities in hospital beds between the central and western regions significantly increased after 2011. This might be explained by two factors. First, there was more demand for health services in the central region due to its large population and enhanced medical security after reform. Second, counties in the central region had a lower chance of misappropriating transfer payments to promote regional economic growth and invested more funds into health compared to the western region. [34,35]. According to Chen et al. [34], hospital transfer payments maybe misappropriated by local governments with insufficient fiscal ability, such as many counties in the western region, when funds are redirected to promote local economic growth due to the western region’s cadres’ evaluation system.

In 2003, China launched the New Cooperative Medical Scheme (NCMS) that saw governments subsidize a health insurance scheme to pool of risks of diseases for rural residents. Recent research found that NCMS has significantly improved the outpatient utilization in county-level hospitals in the eastern and central regions, but had an insignificant impact on rural residents’ outpatient services in the western region [36]. In contrast, they found that the NCMS significantly improved the inpatient utilization in the western region [36]. These different effects on outpatient and inpatient utilization across the three regions may have significantly different effects on the outpatient and inpatient workload in these regions and increased the regional disparities in daily inpatient stays per doctor.

This study has several policy implications. First, more hospitals should build in western region to preserve equity and accessibility to health services given its relatively larger territory. This requires more transfer payments to western region. Second, effective policies should be developed to attract more qualified TCM staff, reducing the doctors’ workload and narrowing regional disparities. Third, targeted health policy with optimized hospitals’ scale should be a priority in shaping China’s TCM system.

This study is subject to several limitations. First, we only focused on the time trends of regional disparities. Although we have discussed some potential reasons for these tendencies, the present study does not support inferences about the effects of specific policy interventions. Second, we are aware that our indicators were restricted by the availability of data. Although our mesures are consistent with other studies [3,11,12], they may not reflect the entire picture of inequality in the TCM county hospitals. For example, no quality outcomes were analyzed in this study and measurements of doctors’ workloadonly focued on the operational efficiency.

Our study also has many strengths. This is the first study to estimate the regional disparities and their time trends in the characteristics of county level TCM hospitals in China, which provides new information about TCM health resource allocation. According to the outline of strategic planning for the development of TCM 2016, each county should have at least one TCM hospital. County level TCM hospitals occupied major health resources and were the main health care providers in China, especially in rural area. Exploring the distribution and changes of these resources provides general insights in TCM health care resource allocation. In addition, we used longitudinal data of all county level TCM hospitals in China between 2004 and 2016, which avoids sampling bias.

## Conclusion

Although TCM county hospital funding and hospital number increased over the past 13 years, substantial regional disparities remain. During the post-reform period, regional disparities in the number of TCM hospitals between the eastern and western regions were corrected to some extent. But changes in hosptals’ scale and doctors’ workload did not address regional disparities for TCM county hospitals. Further, the 2009 reform may have encouraged unreasonable expansion in TCM hospital scale and raised the doctors’ workload of TCM hospitals in the west area, without narrowing regional disparities. This implies that policy measures should be directed to optimizing the number of TCM hospitals, attracting quality TCM doctors and reducing the doctors’ workload, especially in the westen region, rather than promoting the scale up of TCM hospitals.

## Supporting information

S1 Fig Information criteria from 36 (6 outcomes*6 breakpoints) regression models with different breakpoints indicating the best fitting model has a breakpoint at 2011. (TIF)

## Abbreviations

NCMS: New Cooperative Medical Scheme
TCM: traditional Chinese medicine

## References

[1] Fan S, Kanbur R, Zhang X. China’s regional disparities: Experience and policy. Rev Dev Finance. 2011;1: 47–56. 10.1016/j.rdf.2010.10.001

[2] Kanbur SR, Lustig N. Why is inequality back on the agenda? Department of Agricultural, Resource, and Managerial Economics, Cornell University; 1999.

[3] Fang P, Dong S, Xiao J, Liu C, Feng X, Wang Y. Regional inequality in health and its determinants: Evidence from China. Health Policy. 2010;94: 14–25. 10.1016/j.healthpol.2009.08.002 19735959

[4] Murray CJL, Frenk J. A framework for assessing the performance of health systems. Bull World Health Organ. 2000; 15. PMC2560787 10916909

[5] Horev T, Pesis-Katz I, Mukamel DB. Trends in geographic disparities in allocation of health care resources in the US. Health Policy. 2004;68: 223–232. 10.1016/j.healthpol.2003.09.011 15063021

[6] Pan J, Shallcross D. Geographic distribution of hospital beds throughout China: a county-level econometric analysis. Int J Equity Health. 2016;15 10.1186/s12939-016-0467-9 27821181 PMC5100192

[7] Zhang T, Xu Y, Ren J, Sun L, Liu C. Inequality in the distribution of health resources and health services in China: hospitals versus primary care institutions. Int J Equity Health. 2017;16 10.1186/s12939-017-0543-9 28253876 PMC5335774

[8] Liang D, Zhang D, Huang J, Schweitzer S. Does Rapid and Sustained Economic Growth Lead to Convergence in Health Resources: The Case of China From 1980 to 2010. Inq J Health Care Organ Provis Financ. 2016;53: 004695801663169 10.1177/0046958016631699 26895881 PMC5798700

[9] China’s state council. Outline of strategic planning for the development of TCM (2016–2030). 2016 [cited 12 Nov 2019]. http://www.gov.cn/xinwen/2016-02/26/content_5046727.htm

[10] China’s state council. White paper on the development of traditional Chinese medicine (TCM) in China. 2016 [cited 1 Jul 2019]. https://www.scio.gov.cn/32618/Document/1534638/1534638.htm

[11] Lu L, Zeng J. Inequalities in the geographic distribution of hospital beds and doctors in traditional Chinese medicine from 2004 to 2014. Int J Equity Health. 2018;17 10.1186/s12939-018-0882-1 30419919 PMC6233493

[12] Wang L, Suo S, Li J, Hu Y, Li P, Wang Y, et al An investigation Into Traditional Chinese Medicine Hospitals in China: Development Trend and Medical Service Innovation. Int J Health Policy Manag. 2016;6: 19–25. 10.15171/ijhpm.2016.72 28005539 PMC5193503

[13] Guo S, Zou J. Study on Fiscal Transfer Payment System Reform in China—Based on the Perspective of a New Road to Urbanization. Mod Econ. 2015;06: 871–880. 10.4236/me.2015.68082

[14] Wagstaff A, Lindelow M, Wang S, Zhang S. Reforming China’s Rural Health System. The World Bank; 2009.

[15] Bank TW. Deepening health reform in China : building high-quality and value-based service delivery—policy summary. The World Bank; 2016 7 pp. 1–202. Report No.: 107176. http://documents.worldbank.org/curated/en/800911469159433307/Deepening-health-reform-in-China-building-high-quality-and-value-based-service-delivery-policy-summary

[16] Yip WC-M, Hsiao WC, Chen W, Hu S, Ma J, Maynard A. Early appraisal of China’s huge and complex health-care reforms. The Lancet. 2012;379: 833–842. 10.1016/S0140-6736(11)61880-1 22386036

[17] WPRO. Health sector reform in China. In: Health sector reform in China [Internet]. 2011 [cited 12 Dec 2018]. http://www.wpro.who.int/china/mediacentre/factsheets/health_sector_reform/en/

[18] CPC Central Committee and the State. Opinions of the CPC Central Committee and the State Council on Deepening the Health Care System Reform. 2009. http://www.gov.cn/jrzg/2009-04/06/content_1278721.htm

[19] China’s state council. Implementation Plan for the Recent Priorities of the Health Care System Reform (2009–2011) (Guo Fa [2009] NO.12). 2009 [cited 7 Jan 2019]. http://www.gov.cn/zwgk/2009-04/07/content_1279256.htm

[20] Han B. Analysis the development of county-level publich traditional Chinese medicine hospital from 2006 to 2011. Master, Beijing University of Chinese Medicine. 2013.

[21] China’s state council. Opinions of the State Council on supporting and promoting the development of TCM (Guo Fa [2009] NO.22). [cited 7 Jan 2019]. http://www.gov.cn/zwgk/2009-05/07/content_1307145.htm

[22] Jin J, Wang J, Ma X, Wang Y, Li R. Equality of Medical Health Resource Allocation in China Based on the Gini Coefficient Method. Iran J Public Health. 2015;44: 13. PMC4441957 26056663

[23] Zhou K, Zhang X, Ding Y, Wang D, Lu Z, Yu M. Inequality trends of health workforce in different stages of medical system reform (1985–2011) in China. Hum Resour Health. 2015;13 10.1186/s12960-015-0089-0 26645960 PMC4673776

[24] Wang S, Xu J, Jiang X, Li C, Li H, Song S, et al Trends in health resource disparities in primary health care institutions in Liaoning Province in Northeast China. Int J Equity Health. 2018;17 10.1186/s12939-018-0896-8 30514300 PMC6280446

[25] He C, Liu L, Chu Y, Perin J, Dai L, Li X, et al National and subnational all-cause and cause-specific child mortality in China, 1996–2015: a systematic analysis with implications for the Sustainable Development Goals. Lancet Glob Health. 2017;5: e186–e197. 10.1016/S2214-109X(16)30334-5 28007477 PMC5250590

[26] Zhou M, Wang H, Zhu J, Chen W, Wang L, Liu S, et al Cause-specific mortality for 240 causes in China during 1990–2013: a systematic subnational analysis for the Global Burden of Disease Study 2013. The Lancet. 2016;387: 251–272. 10.1016/S0140-6736(15)00551-6 26510778

[27] Division of the Eastern, Central and Western China. China Popul Today. 2000; 3.

[28] Keppel K, Pamuk E, Lynch J, Carter-Pokras O, Kim I. Methodological Issues in Measuring Health Disparities. Vital Health Stat. 2013; 34. PMC3681823 16032956

[29] Barr B, Higgerson J, Whitehead M. Investigating the impact of the English health inequalities strategy: time trend analysis. BMJ. 2017; j3310. 10.1136/bmj.j3310 28747304 PMC5527348

[30] National Health and Family Planning Commission. China Health and family planning Yearbook. China union medical university press; 2016.

[31] Wu Q, Ma X, Chen L, Yang Y. SWOT Analysis of Personnel Filing System in Public Hospitals. Chin Hosp Manag. 2016;36: 11–13.

[32] Chen L, Ma X, Huang Y. Historical Evolution of Public Hospital Establishment System Reform from Institutional Change Angle. Chin Hosp Manag. 2016;36: 1–3.

[33] Jiang S, Min R, Fang P. The impact of healthcare reform on the efficiency of public county hospitals in China. BMC Health Serv Res. 2017;17 10.1186/s12913-017-2780-4 29262816 PMC5738802

[34] Chen T, Wang Y, Luo X, Rao Y, Hua L. Inter-provincial inequality of public health services in China: the perspective of local officials’ behavior. Int J Equity Health. 2018;17 10.1186/s12939-018-0827-8 30064429 PMC6069573

[35] Li H, Zhou L-A. Political turnover and economic performance: the incentive role of personnel control in China. J Public Econ. 2005;89: 1743–1762. 10.1016/j.jpubeco.2004.06.009

[36] Liu D, Tsegai D, Litaker D, von Braun J. Under regional characteristics of rural China: a clearer view on the performance of the New Rural Cooperative Medical Scheme. Int J Health Econ Manag. 2015;15: 407–431. 10.1007/s10754-015-9175-z 27878696

