# Supplementary figures and images for: Regional disparities in health care resources in traditional Chinese medicine county hospitals in China
Source: PLoS One. 2020 Jan 21;15(1):e0227956. doi: 10.1371/journal.pone.0227956 (PMC6974170; doi:10.1371/journal.pone.0227956)

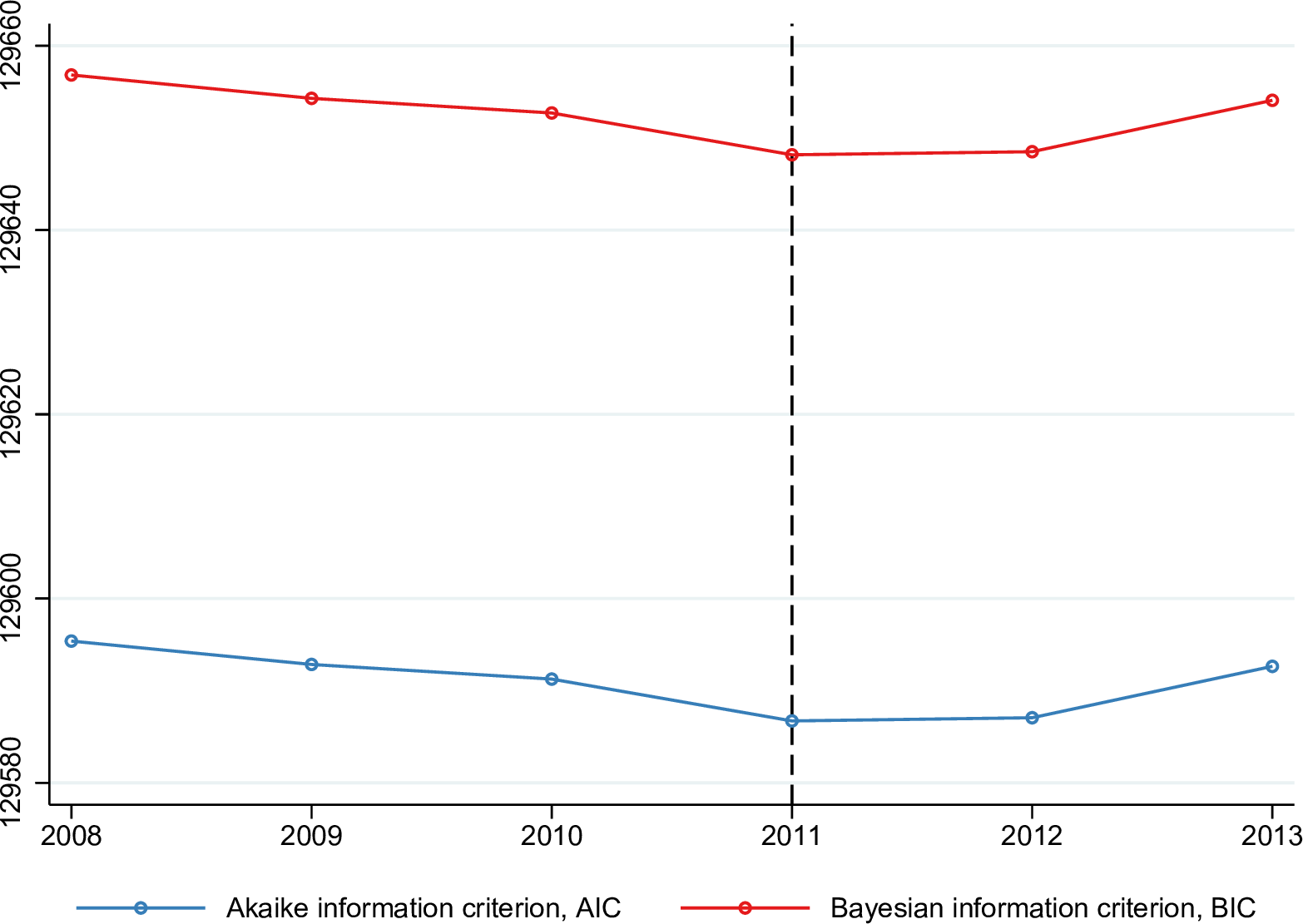

Supplement: S1 Fig — (TIF) [file pone.0227956.s001.tif]
